# Classification schemes for knowledge translation interventions: a practical resource for researchers

**DOI:** 10.1186/s12874-017-0441-2

**Published:** 2017-12-06

**Authors:** Susan E. Slaughter, Gabrielle L. Zimmermann, Megan Nuspl, Heather M. Hanson, Lauren Albrecht, Rosmin Esmail, Khara Sauro, Amanda S. Newton, Maoliosa Donald, Michele P. Dyson, Denise Thomson, Lisa Hartling

**Affiliations:** 1 University of Alberta, Edmonton, Canada; 2 Alberta SPOR SUPPORT Unit KT Platform, Edmonton, Canada; 3 University of Calgary, Calgary, Canada; 4 Alberta Health Services, Calgary, Canada

**Keywords:** Knowledge translation interventions, Classification schemes, AGREE II tool, Healthcare, Implementation science

## Abstract

**Background:**

As implementation science advances, the number of interventions to promote the translation of evidence into healthcare, health systems, or health policy is growing. Accordingly, classification schemes for these knowledge translation (KT) interventions have emerged. A recent scoping review identified 51 classification schemes of KT interventions to integrate evidence into healthcare practice; however, the review did not evaluate the quality of the classification schemes or provide detailed information to assist researchers in selecting a scheme for their context and purpose. This study aimed to further examine and assess the quality of these classification schemes of KT interventions, and provide information to aid researchers when selecting a classification scheme.

**Methods:**

We abstracted the following information from each of the original 51 classification scheme articles: authors’ objectives; purpose of the scheme and field of application; socioecologic level (individual, organizational, community, system); adaptability (broad versus specific); target group (patients, providers, policy-makers), intent (policy, education, practice), and purpose (dissemination versus implementation). Two reviewers independently evaluated the methodological quality of the development of each classification scheme using an adapted version of the AGREE II tool. Based on these assessments, two independent reviewers reached consensus about whether to recommend each scheme for researcher use, or not.

**Results:**

Of the 51 original classification schemes, we excluded seven that were not specific classification schemes, not accessible or duplicates. Of the remaining 44 classification schemes, nine were not recommended. Of the 35 recommended classification schemes, ten focused on behaviour change and six focused on population health. Many schemes (*n* = 29) addressed practice considerations. Fewer schemes addressed educational or policy objectives. Twenty-five classification schemes had broad applicability, six were specific, and four had elements of both. Twenty-three schemes targeted health providers, nine targeted both patients and providers and one targeted policy-makers. Most classification schemes were intended for implementation rather than dissemination.

**Conclusions:**

Thirty-five classification schemes of KT interventions were developed and reported with sufficient rigour to be recommended for use by researchers interested in KT in healthcare. Our additional categorization and quality analysis will aid in selecting suitable classification schemes for research initiatives in the field of implementation science.

**Electronic supplementary material:**

The online version of this article (10.1186/s12874-017-0441-2) contains supplementary material, which is available to authorized users.

## Background

With the advancement of implementation science, knowledge translation (KT) interventions to promote the translation of research evidence into practice are increasing considerably. KT interventions can target different levels such as health providers (e.g., reminders to complete a new health assessment), health systems (e.g., introduction of a new form to facilitate documentation) and health policy (e.g., reimbursement scheme to encourage a new practice). With the growth in KT interventions, taxonomies or classifications schemes have begun to emerge to help clarify details, promote consistency in reporting, and facilitate an understanding of the interventions.

A recent scoping review by Lokker et al. identified 51 diverse classification schemes of interventions to promote and integrate evidence into healthcare practice [1]. The review provides researchers with a high level overview of schemes to classify KT interventions with the intent to address challenges of detailed describing and reporting of interventions. One important limitation to this review is the paucity of information to guide researchers in selecting a particular scheme suitable for their context and purpose. Guidance exists for selecting models, theories, and frameworks to assist with interpretation of study fındings and to ensure the inclusion of essential implementation strategies [2, 3]. For example, one recent narrative review identified 41 different conceptual frameworks to describe and measure key elements of the process for translating research evidence into policy and practice [4]. Another narrative review identified 61 theories and models to provide a systematic way of understanding, developing and evaluating dissemination and implementation research [2]. Furthermore, an interactive website exists to help researchers and practitioners select the dissemination and implementation model that best fits their research question or practice problem [3]. While this guidance can help direct development, selection or evaluation of KT interventions [2–4], it does not provide guidance for consistent description of KT interventions. Both are important to improve the reporting and generalizability of KT interventions.

Lokker et al. acknowledged that additional work is needed to be able to apply these classification schemes in an optimal and meaningful way by researchers [1]. Furthermore, critical appraisal or quality assessment of the classification schemes would provide important information on the rigour of the schemes’ development. To address these gaps, the Knowledge Translation Methods Working Group, which is an initiative of the Knowledge Translation Platform of the Alberta Strategy for Patient Oriented Research (SPOR) SUPPORT Unit, undertook a more in-depth analysis of the classification schemes identified by Lokker et al. The purpose of this study was to examine the classification schemes in more detail, extract additional information, and assess the developmental and methodological quality of each, in order to guide researchers to the tool that might be most appropriate for their specific purpose and context.

## Methods

### General approach

The Alberta SPOR SUPPORT Unit’s Knowledge Translation Platform established a working group involving platform staff, as well as academics, trainees, and health service employees across the province with an interest in KT science. Through discussion and consensus, the working group identified an in-depth analysis of the classification schemes for the implementation of evidence into healthcare as a priority area within KT. The group met biweekly (on average) over the course of a year, and collectively developed the project scope and methods, collected and analyzed data, discussed findings, developed consensus and drafted the final report.

### Description of classification schemes

#### Data collection

The original paper [1] provided very general information about each scheme including: sorting schemes as lists, taxonomies, frameworks, or ‘other’; reporting context, focus and brief methodological notes; and indicating if the scheme had been peer reviewed, involved knowledge users in its development, was piloted or tested, or was theory based. The working group members independently extracted the following additional information: authors’ objectives; purpose of the classification scheme; field of application (e.g., public health, tobacco control, mental health); adaptability (i.e., broad versus specific); whether the interventions targeted patients, providers, or policy makers; socioecologic level; dissemination versus implementation; and focus of implementation, referred to as intent. Through consensus, the working group identified these additional data as useful in the selection of a classification scheme for use in KT research.

Adaptability of schemes, socioecologic level, and dissemination versus implementation were adapted from a previous review of models for dissemination and implementation research [2]. Adaptability of schemes was categorized as broad versus specific, relative to the application and/or operationalization of the classification scheme [2]. Socioecologic level, defined as level of influence, was categorized as individual, organization, community, and system [2]. In addition to these structural levels of influence, we sought to identify which specific target group the interventions within the classification schemes applied to (i.e. patients, providers or policy makers). Focus of implementation, or intent, was categorized as clinical practice, education, or policy. Socioecologic level and intent categories were not mutually exclusive (i.e., a given classification scheme could have more than one). As per the definitions provided by Tabak et al., dissemination is “focus on active approach of spreading evidence-based interventions to the target audience via determined channels using planned strategies” whereas implementation is “focus on process of putting to use or integrating evidence-based interventions within a setting” [2]. We also abstracted information on methodology, including whether the scheme was peer-reviewed, involved knowledge users in its development, was piloted or tested, and was theory based, in order to provide a comprehensive description of each [1]. Extracted data were independently assessed by a second reviewer during quality appraisal. Both reviewers also independently assessed the utility of each classification scheme.

In order to gain an understanding of the use of each classification scheme, each article was searched in Scopus by title. The total number of citations in the past five years, the subject area of the citations (as defined by Scopus, e.g., medicine, psychology, nursing), and the document type (e.g., articles, reviews, conference papers) were recorded.

#### Quality appraisal

In the absence of an established tool to appraise the methodological quality of the classification schemes, we used the AGREE II tool as the basis for developing our appraisal tool. The AGREE II tool is well recognized, has been rigorously developed and covers many aspects of quality relevant to classification schemes [5]. The AGREE II tool was designed to be applied to clinical practice guidelines; therefore, we adapted the tool to make the items relevant to KT classification schemes. All working group members were involved in making the adaptations based on an iterative process of applying the tool to a sample of articles, and discussing challenges and appropriateness of the items. In this way, the working group, made up of relevant knowledge users, was able to provide face validity and initial content validity for the adapted tool to assess quality of KT classification schemes. The adapted AGREE II tool includes six domains that are each scored on a seven-point Likert scale (strongly disagree = 1 to strongly agree = 7). The overall score, in both the original and our adapted version, is *not* based on a mathematical computation of the domain scores. Instead, the score, which ranges from 1 (lowest possible quality) to 7 (highest possible quality), is based on overall impressions of the classification scheme, which take the six domains into account. An additional file shows the adapted domains of the AGREE II tool [see Additional file 1]. The 12 members of the working group were randomly assigned to conduct a quality appraisal of the articles such that two people independently assessed each article and the pair arrived at consensus for each domain and overall rating. In this way each member worked with several others; this helped identify questions or areas of discrepancy which were brought to the full working group for discussion, then decision rules were established to ensure consistency. These decision rules were sufficient to eliminate any further major discrepancies in ratings (and allowed the reviewers to come to within 2 points and/or achieve consensus easily?). In accordance with AGREE II guidance, classification schemes were recommended for use, or not recommended for use by the pair of working group members reviewing the classification scheme, based on the quality score, in conjunction with their overall impression of the classification schemes’ utility. All classification schemes that were not recommended were discussed with the full working group. Members of the working group, comprising researchers from academia and health services, who are potential knowledge users of these classification schemes, were able to assess the utility of the classification schemes for intended users.

#### Data analysis

We summarize our findings using descriptive statistics. To understand the context around previous use of the classification schemes, independent sample t-tests were used to compare the years of publication and number of Scopus citations per year since publication of the recommended and not recommended classification schemes.

## Results

### Recommended classification schemes

**Fig. 1:**
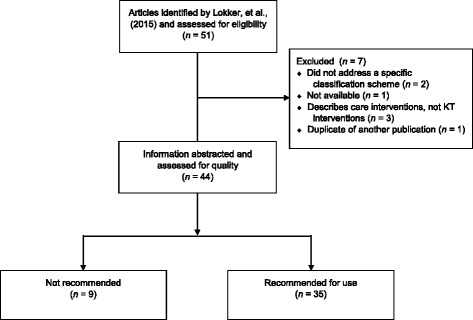
Article flow diagram

Figure 1 illustrates the flow of the articles within our study. During our initial data extraction, we excluded seven articles that had originally been included by Lokker et al. [1]. One was a website that was no longer active [6]; two were textbooks that provided general information but not a specific classification scheme [7, 8]; three did not describe a specific KT classification scheme [9–11]; and one was considered a duplicate [12].

After excluding articles that were not accessible, duplicates, or not focused on a specific KT classification scheme, we included 44 articles in our descriptive analysis and quality appraisal. Nine articles [13–21] were assessed to be of low quality, or did not provide schemes that were deemed useful for KT researchers as judged by our working group of knowledge users. Table 1 summarizes details of the articles containing classification schemes that were not recommended.

**Table 1:** Classification schemes of KT interventions that were not recommended for use

Article	Purpose of Classification Scheme	Area of Application	Citations^a^	Quality Score^b^	Rationale for Not Recommending
Cohen 2000 [13]	To outline and clarify the content of preventative interventions	STD/HIV prevention	27	3	Low scores across all domains, modification of existing tool with minimal detail on development
Dolan 2010 [14]	To shape policy maker behaviour	Policy	254	4	Poor rigour of development; does not seem useful for researchers
Embry 2008 [15]	To provide a database repository of evidence-based units of behavioural influence	Parenting, school, & public health behaviour	88	4	Poor rigour of development and applicability, not intended as a development document, creates a care-focused sample
Geller 1990 [16]	To outline a conceptual framework for traffic safety, especially use of seat belts	Injury prevention	51	3	Complex and outdated; poor rigour of development, applicability and KU involvement
Goel 1996 [17]	To outline influences on retail pharmacies in developing countries	Pharmacy behaviours	114	3	Low scores in rigour of development, applicability, and editorial independence
Hardeman 2000 [18]	To describe behaviour change programmes	Population/ public health	178	2	Low scores for scope and purpose, stakeholder/KU involvement, aspects of rigour, and applicability
Perdue 2005 [19]	To describe different legal strategies for chronic disease prevention	Policy & public health	17	3	Low scores in areas of rigour of development and applicability
Reisman 2005 [20]	To provide a taxonomy for transfer of technology	Technology transfer	36	3	Low scores in rigour of development, applicability, and editorial independence
West 2006 [21]	To review tobacco control strategies	Behaviour change	28	2	Low scores for scope and purpose, stakeholder/KU involvement, aspects of rigour, and applicability

^a^Number of citations for each article via title search in Scopus

^b^Quality score is the overall adapted AGREE II score, reached by consensus between two researchers

Thirty-five articles [22–56] were recommended. A description of the recommended classification schemes can be found in Table 2. In most cases the quality appraisals aligned with the recommendations; however there were four exceptions. Two low-scoring classification schemes were recommended [26, 49], and two intermediate-scoring classification schemes were not recommended [14, 15]. The low-scoring classification schemes were assigned a two and a three because little to no information was provided about how the classification schemes were developed. These two schemes classified interventions to implement evidence into healthcare involving reimbursement schemes [26] and public health policy initiatives, [49] respectively. We opted to recommend them because, in the absence of any other classification schemes for these areas of healthcare, they offer unique and potentially useful contributions to the KT literature. Conversely, the classification schemes reported by Embry et al. [15] and Dolan et al. [14] both scored a four; however we did not recommend these classification schemes. The paper by Embry et al. [15] did not focus on a classification scheme for interventions to implement evidence into healthcare; rather it focused on behavioral prevention and treatment practices and only a partial classification scheme was presented [15]. Dolan et al. provided a checklist of influences on behaviour that should be considered in public policy making [14]; the checklist received low scores in rigour of development which reflected the assessment that it was not a useful classification scheme for KT researchers.

**Table 2:** Classification schemes of KT interventions that were recommended for use

Article	Purpose of Classification Scheme	Area of Application	Context of Previous Use^a^	Number of Citations^b^	Quality Score^c^
Abraham 2008 [22]	To provide a common vocabulary for behaviour change interventions	Behaviour change	Physical activity, healthy eating, change in cognition, HIV/AIDS	816	6
Albrecht 2013 [23]	To compare quality of reporting and types of KT interventions being used	Behaviour change	–	44	5
Best 2008 [24]	To improve past Cancer Control Frameworks	Cancer practice & policy	Cancer practice & policy	54	4
Cane 2012 [25]	To “simplify and integrate” multiple behaviour change theories, by refining the theoretical domains framework (TDF)	Behaviour change	–	259	6
Carlson 2010 [26]	To categorize future health outcomes-based reimbursement schemes	Reimbursement schemes	–	107	2
Century 2012 [27]	To understand (1) aspects of implementation, (2) factors that affect implementation, and (3) tools for measuring these	Education	–	9	5
CIHI 2001 [28]	To summarize strategies by target audience, timing and methods	Population health	Health policy & decision making	Not found	4
Czaja 2003 [29]	Taxonomy of complex psychosocial and behaviour interventions	Alzheimer’s disease	Alzheimer’s disease	42	5
Damschroder 2009 [30]	List of constructs to promote theory development and verification across multiple settings	Multiple	–	1101	6
Dixon 2010 [31]	To describe competency domains for health behaviour change interventions	Behaviour change	Public health	8	6
Dogherty 2010 [32]	Taxonomy of facilitation interventions/strategies and facilitator role synopsis	Nursing implementation	Nursing	50	4
Dy 2011 [33]	To classify patient safety practices	Patient safety	Patient safety	12	6
EPOC 2010 [34]	To (1) help authors register a title with EPOC; and (2) address key issues that frequently arise in EPOC protocols and reviews in the background and methods section	Health care	–	Not found	6
Galbraith 2011 [35]	To identify elements of behavioural interventions that guide translation of interventions from research to practice	HIV/AIDS prevention	HIV/AIDS Prevention	20	6
Gifford 2013 [36]	To inform future research about leadership behaviour	Nursing	Nursing	18	6
Greenhalgh 2004 [37]	To use for diffusion of innovations in health services organizations	Health services	–	2207	5
Hendriks 2013 [38]	To facilitate action-oriented approach for policy makers addressing wicked problems	Population health	Population health & health policy	16	5
Keller 2004 [39]	To identify and document interventions for public health nurses	Public health	Public health nursing	68	6
Lamb 2011 [40]	Taxonomy of interventions used to prevent falls in older adults	Geriatric medicine	Injury prevention & geriatric medicine	34	5
Lavis 2006 [41]	To inform national level dialogue on linking research to action	Knowledge translation	–	171	5
Leeman 2007 [42]	Taxonomy categorizing implementation methods	Nursing	–	48	4
Lowe 2011 [43]	Taxonomy of interventions to improve consumers’ medicines	Patient safety; behaviour change	Prescribing practices	9	6
Mazza 2013 [44]	Taxonomy to classify the nature and content of implementation strategies	Implementation science	–	19	4
Michie 2011a [45]	To link interventions to potential behavioural targets	Behaviour change	Tobacco control & obesity	557	7
Michie 2011b [46]	To provide basis for improving reliable and systematic application of evidence and theory for interventions	Behaviour change	Physical activity & healthy eating	345	6
Michie 2011c [47]	Taxonomy of behaviour change techniques for smoking cessation	Behaviour change	Smoking cessation & health promotion	114	6
Michie 2012 [48]	To identify behaviour change techniques used to reduce excessive alcohol consumption	Behaviour change	Reduction of alcohol consumption	64	6
Nuffield 2007 [49]	To justify different policy initiatives in public health	Public health	Infectious disease, obesity, smoking/alcohol & water fluoridation	Not found	3
Powell 2012 [50]	To provide implementation strategies for innovations	Mental health	Mental health	117	6
Schulz 2010 [51]	To assess the relationships between outcomes and intervention components	Implementation science	Implementation science	45	6
Shojania 2004 [52]	To help users assess whether evidence suggests that a quality improvement strategy is applicable to their context	Quality improvement	Multiple disease areas	Not found	6
Stirman 2013 [53]	To classify modifications to evidence-based programs during implementation	Implementation science	–	40	6
Taylor 2011 [54]	To categorize contextual features influencing successful implementation	Patient safety	–	66	6
Walter 2003 [55]	To increase the impact of research	Policy & behaviour change	–	45	4
Ward 2010 [56]	To improve success of incorporating research-based knowledge into action	Knowledge translation	–	71	6

^a^Area where scheme has previously been tested. Those with – have not been tested in any specific context

^b^Number of citations for each article via title search in Scopus

^c^Quality score is the overall adapted AGREE II score, reached by consensus between two researchers

There was a significant difference in the year of publication for the classification schemes recommended and not recommended; recommended classification schemes were more recently published (*Mean* = 2009, *SD* = 3.3) than schemes not recommended (*Mean* = 2002, *SD* = 6.3; *p* < 0.05). We also found a significant difference in the number of citations normalized over the years since publication between the recommended (*Mean* = 24.8, *SD* = 41.4) and not recommended (*Mean* = 8.0, *SD* = 11.1), *p* < 0.05) classification schemes (*p* < 0.05).

### Field of application and adaptability of schemes

We assessed the field of application for each recommended classification scheme. Ten schemes focused on behaviour change [22, 23, 29, 31, 43, 45–48, 55], six focused on population health [28, 38–41, 49], and 19 schemes had general applicability [24–27, 30, 32–37, 42, 44, 50–54, 56]. Table 3 summarizes details for all recommended schemes.

**Table 3:** Details of recommended classification schemes for KT interventions

Article	Adaptability of Schemes B = Broad S = Specific	Level of Influence				Intent			Implementation vs Dissemination I = Implementation D = Dissemination	Target Group PT = Patient PR = Provider PM = Policy makers
		Individual	Organization	Community	System	Policy	Education	Practice		
Behaviour Change										
Abraham 2008 [22]	B	•						•	I	PT
Albrecht 2013 [23]	B	•	•	•	•		•	•	I & D	PR
Czaja 2003 [29]	B & S	•	•	•	•		•	•	I	PT & PR
Dixon 2010 [31]	B	•	•			•	•	•	I & D	PT & PR
Lowe 2011 [43]	B	•			•	•	•	•	I	PT
Michie 2011a [45]	B & S	•	•		•	•		•	I	PT & PR
Michie 2011b [46]	S	•	•		•			•	I	PT & PR
Michie 2011c [47]	S	•	•		•	•		•	I	PT & PR
Michie 2012 [48]	B & S	•	•		•	•		•	I	PR
Walter 2003 [55]	B	•	•	•	•	•		•	I & D	PR
Population Health										
CIHI 2001 [28]	B		•	•	•	•			D	PR
Hendriks 2013 [38]	B				•	•			I	PM
Keller 2004 [39]	B	•		•	•			•	I	PR
Lamb 2011 [40]	S		•					•	I	PR
Lavis 2006 [41]	B				•	•		•	I & D	PR
Nuffield 2007 [49]	B				•	•			I	PR
General										
Best 2008 [24]	B & S	•	•	•	•	•	•	•	I & D	PT & PR
Cane 2012 [25]	B	•						•	I	PR
Carlson 2010 [26]	B		•		•	•			I	PR
Century 2012 [27]	B		•		•		•		I	PR
Damschroder 2009 [30]	B		•	•	•	•	•	•	I	PR
Dogherty 2010 [32]	B	•	•					•	I	PR
Dy 2011 [33]	S		•	•	•	•	•	•	I	PR
EPOC 2010 [34]	B	•	•	•	•	•	•	•	I & D	PT & PR
Galbraith 2011 [35]	B	•		•			•	•	I	PR
Gifford 2013 [36]	S	•	•					•	I	PR
Greenhalgh 2004 [37]	B	•	•	•	•	•	•	•	I & D	PR
Leeman 2007 [42]	B	•	•					•	I	PR
Mazza 2013 [44]	B				•	•			I	PR
Powell 2012 [50]	B		•		•			•	I	PR
Schulz 2010 [51]	S	•	•					•	I	PR
Shojania 2004 [52]	B	•	•	•	•	•	•	•	I & D	PT & PR
Stirman 2013 [53]	B	•	•	•		•	•	•	I	PR
Taylor 2011 [54]	B	•	•					•	I	PT & PR
Ward 2010 [56]	B		•	•	•	•		•	I	PR

We also categorized the intent of the intervention of the included schemes within the domains of practice, education, and/or policy. Many schemes (*n* = 29) addressed practice considerations [22–25, 29–37, 39–43, 45–48, 50–56]. Fewer schemes addressed educational (*n* = 13) [23, 24, 27, 29–31, 33–35, 37, 43, 52, 53] and policy (*n* = 20) objectives [24, 26, 28, 30, 31, 33, 34, 37, 38, 41, 43–45, 47–49, 52, 53, 55, 56].

The adaptability of each classification scheme was assessed as either broad (defined as greater flexibility to apply the scheme to a wide array of contexts/clinical areas) or specific (defined as the scheme being developed for a specific context/clinical area). The vast majority of schemes had broad adaptability (*n* = 25) [22, 23, 25–28, 30–32, 34, 35, 37–39, 41–44, 49, 50, 52–56]. In contrast, six schemes were identified as specific, offering detailed actions for dissemination and implementation [33, 36, 40, 46, 47, 51]. The remaining four schemes included elements of both broad and specific adaptability [24, 29, 45, 48].

### Level of influence of schemes

There was diversity in the socioecologic level or level of influence of the classification schemes. Seven of the 35 schemes targeted a single level while the remaining 28 schemes targeted two or more levels. The level least targeted within the schemes was the community level, such as neighbourhoods or local governments (*n* = 14) [23, 24, 28–30, 33–35, 37, 39, 52, 53, 55, 56]. The individual level, organizational level, and system levels were roughly equally targeted, with 23–26 schemes addressing each of these levels.

### Implementation and dissemination attributes of schemes

The focus of the schemes was predominantly implementation activities focused on the process of using evidence within the given setting (*n* = 26) [22, 25–27, 29, 30, 32, 33, 35, 36, 38–40, 42–51, 53, 54, 56]. Eight schemes focused on both implementation and dissemination activities [23, 24, 31, 34, 37, 41, 52, 55], and one scheme focused on dissemination only [28].

### Target group

The most frequent group targeted by the classification scheme was healthcare or service providers (*n* = 23) [23, 25–28, 30, 32, 33, 35–37, 39–42, 44, 48–51, 53, 55, 56]. Nine schemes targeted both patients/clients and providers [24, 29, 31, 34, 45–47, 52, 54], two schemes targeted patients/clients alone [22, 43], and one scheme targeted policy makers [38].

## Discussion

This in-depth review delivers key information on a diverse set of classification schemes of interventions for implementing evidence into healthcare, providing a needed resource for researchers to select a classification scheme most appropriate for their purpose and setting. With a dearth of evidence to guide the selection of the most appropriate framework(s) for specific contexts and purposes [57], this study builds on previous work, and broadly categorizes the classification schemes as recommended or not recommended. The results of this project have confirmed the availability of diverse classification schemes for interventions to implement evidence into healthcare, but with variable quality. Notably, there was substantial growth in the publication of classification schemes beginning in 2010. Twenty-three of the classification schemes included in this project were published between 2010 and 2013, compared to 12 published in the preceding nine years. This growth coincides with the advancement of the field, which began in earnest in the mid-1990s and has rapidly expanded [58, 59]. Scientific advancement has led to a dramatic increase in published research and initiated calls for improved methodological rigour within implementation science [60–64]. This, in turn, has led to the publication of frameworks and tools to support the development, implementation, evaluation, and reporting of KT research [1, 2, 65]. However, up until now, there has not been a consistent method developed to assess the quality or methodological rigour of these frameworks and tools. We found that the rigour of development varied among the schemes, with many low scores, which further supports the need to increase the rigour, transparency and credibility of these classification schemes as well as other frameworks and tools. Overall, ‘recommended’ classification schemes demonstrated higher quality scores. Recommended classification schemes had significantly more recent publication dates compared to schemes that were not recommended. They also had more citations per year since publication than did the classifications schemes not recommended. These findings are likely due to the advancements made in the rigour of KT as a science in recent years.

Of the recommended classification schemes, a factor of particular interest was adaptability. Specific classification schemes have been tested in or applied to specific situations, and can offer a ‘grab-and-go’ solution, provided the purpose of the scheme aligns with researchers’ goals and context. For example, Michie and colleagues specifically developed classification schemes (also referred to as taxonomies) for physical activity, healthy eating behaviours [46] and smoking cessation [47]. In contrast, the initial taxonomy/list of behaviour change techniques developed by Abraham and Michie [22] was intended to be more broadly applicable. In this manner, the broad adaptability definition enables greater flexibility to adapt the classification scheme to specific activities and/or contexts, which is especially important if no relevant specific scheme exists. In fact, the majority of the highly cited (i.e., more than 100 citations) ‘recommended’ classification schemes demonstrated broad adaptability (*n* = 8/10); however, questions remain about how to best select from and adapt similar broad classification schemes. For those classification schemes that were labeled both broad and specific, some were first identified as being broadly applicable but were also specifically tested in a given area, which provides a starting point for those researchers who might be working in that area [45, 48]. Others were originally identified as being developed for a specific context or using a specific population, but are described as having broader applicability in other clinical areas [24, 29].

Many of these classification schemes are linked, either by the authors working together or by extending existing schemes [1]. For example, three of the papers by Michie et al. started with the behaviour change techniques (BCTs) identified in the Abraham and Michie paper [22] and produced a more specific/tailored scheme for a particular clinical context (e.g., smoking cessation) [46–48]. Mazza et al. [44] and Shojania et al. [52] each started with, and built upon, the Effective Practice and Organization of Care (EPOC) taxonomy, but unlike the papers by Michie et al., they did not result in a more specific scheme; they are both still categorized as having broad adaptability. Powell et al. [50] built upon Damschroder’s Consolidated Framework for Implementation Research (CFIR) [30], and these authors together (with others) expanded it yet again in 2015 [66]. Schulz et al. [51] built upon the work done by Czaja et al. [29] (both authors are on both papers). If a researcher is interested in adapting or extending a current scheme, it would be worthwhile to see what has already been done or start with one of the current broad schemes that others have found to be a valuable starting point (e.g., EPOC and the BCTs).

Other key factors to guide selection of best classification schemes include field of application, areas in which the tool has been tested, whether it is specific to implementation or dissemination, target group, socioecologic level, and intent (i.e., policy, education, practice). Future research should describe the selection and evaluation of specific KT intervention classification schemes to illuminate the decision-making process, pros and cons of the classification scheme in practice, and any necessary adaptations required to use the tool in a specific context. This would help contextualize the assessment and categorizations presented here and clarify whether these variables are important for researchers when making these decisions.

Nine classification schemes were ‘not recommended’, scoring ≤4 out of 7. These low scores reflected lack of rigour in tool development, limited applicability, and issues related to lack of stakeholder involvement and editorial independence. Eight of nine classification schemes designated ‘not recommended’ were published before the year 2010 and before the rapid accumulation of KT guidance with improved methodological rigour caused older constructs to become outdated. At present, three of the ‘not recommended’ classification schemes are highly cited (i.e., more than 100 citations) [14, 17, 18], leading to questions of de-adoption. De-adoption refers to the process of discontinuing a health practice, service, intervention that has been shown to be ineffective [67]. Future research should ascertain whether these classification schemes are of low value in all fields, explore mechanisms to reduce use, and evaluate effectiveness and sustainability of de-adoption strategies [68].

Using the same cohort of classification schemes for KT interventions, an international team recently created a new overarching KT schema (Aims, Ingredients, Mechanism, Delivery [AIMD] framework) [57, 69]. The AIMD framework is specific to the development and reporting of KT interventions and proposed as an easy-to-use tool to reduce the ‘noise’ from the litany of available classification schemes and standardize terminology. Preliminary pilot testing and validation work have demonstrated promising results [57]; however, AIMD has yet to be experimentally evaluated and it does not incorporate additional, key implementation factors, such as context. Future research evaluating the AIMD framework and comparing it to existing classification schemes would help elucidate the path forward for KT science.

### Strengths and limitations

This study has several strengths. First and foremost, this is a user driven study. A group of multidisciplinary researchers and knowledge users converged to become a team focused on offering practical guidance to select a classification scheme for implementation of evidence into healthcare. Together we identified a need for further research to provide more detailed information necessary to guide the practical work of KT researchers. The application of a quality appraisal tool, to ascertain the methodological rigour of these schemes, has not been conducted previously and provides a novel and meaningful method for categorizing and assessing them.

This study also has limitations. The literature was not systematically searched for an updated, expanded set of classification schemes; instead, we assessed the classification schemes identified by Lokker and colleagues [1]. Critical appraisal of the quality of classification schemes was assessed using an adapted version of the AGREE II tool; while the adapted tool has face and content validity it has not yet been tested for reliability or construct validity. To our knowledge, our adaptation of the AGREE II tool is the only resource available for critical appraisal of KT intervention classification schemes. Future research could assess the adapted tool’s psychometric properties.

## Conclusion

This study reviewed the previously published classification schemes of KT interventions to promote and integrate evidence into healthcare practice, and provided a quality appraisal of these schemes. We extracted additional information from included articles of a scoping review, assessed the developmental and methodological quality of each and made recommendations. Our examination identified 35 classification schemes that may be applicable for researchers and other stakeholders interested in KT in healthcare. We anticipate that our additional categorization and quality appraisal will serve as a practical resource for researchers by facilitating the selection of suitable classification schemes for the researchers’ context and purpose.

## Additional file

Additional file 1: Adaptations of the AGREE II tool. (DOCX 21 kb)

## Abbreviations

AIMD: Aims, Ingredients, Mechanism, Delivery
BCTs: Behaviour change techniques
CFIR: Consolidated Framework for Implementation Research
EPOC: Effective Practice and Organization of Care
KT: Knowledge translation
SPOR: Strategy for Patient Oriented Research
