# Supplementary material for: Classification schemes for knowledge translation interventions: a practical resource for researchers
Source: BMC Med Res Methodol. 2017 Dec 6;17:161. doi: 10.1186/s12874-017-0441-2 (PMC5718087; doi:10.1186/s12874-017-0441-2)
Supplement: Additional file 1: — Adaptations of the AGREE II tool. (DOCX 21 kb) [file 12874_2017_441_MOESM1_ESM.docx]

| **Original AGREE II** | **Adapted AGREE II** | **Rationale for adaptation** |
| --- | --- | --- |
| **Domain 1. Scope and Purpose** | **Domain 1. Scope and Purpose** |  |
| 1. The overall objective(s) of the guideline is (are) specifically described.  2. The health question(s) covered by the guideline is (are) specifically described.  3. The population (patients, public, etc.) to whom the guideline is meant to apply is specifically described. | 1a. The overall objective(s) of the paper is (are) specifically described.  1b. The overall objective(s) of the classification scheme is (are) specifically described.  2. The field(s) of application covered by the classification scheme is (are) specifically described.  3. The population (patients, providers, etc.) to whom the classification scheme is meant to apply is specifically described.  Comments: | We wanted to distinguish between the objectives of the paper versus the objectives of the classification scheme.  The field of application (e.g., behaviour change) was more relevant to the scope and purpose of classification schemes than specific health questions.  We were interested in knowing to whom the classification schemes would be applied.  E.g., Did the classification schemes focus on KT interventions targeted to patients (e.g., decision aids to access health services) or providers (e.g., reminders to complete a health assessment)? |
| **Domain 2. Stakeholder Involvement** | **Domain 2. Stakeholder/Knowledge User Involvement** |  |
| 4. The guideline development group includes individuals from all relevant professional groups.  5. The views and preferences of the target population (patients, public, etc.) have been sought.  6. The target users of the guideline are clearly defined. | 4. All relevant stakeholders/knowledge users were included in the process of developing the classification scheme.  5. The target users of the classification scheme are clearly defined. | Lokker et al originally identified “use of knowledge users in development” as a data element, so we incorporated this as well. Although we felt that it was important to consider the views of those who might be using the classification scheme; we felt it was less important to consider the views of the target population of the interventions.  We were interested in knowing if a given scheme was meant to be used by researchers (our target audience) or policy makers, providers, etc. |
| **Domain 3. Rigour of Development** | **Domain 3. Rigour of Development** |  |
| 7. Systematic methods were used to search for evidence.  8. The criteria for selecting the evidence are clearly described.  9. The strengths and limitations of the body of evidence are clearly described.  10. The methods for formulating the recommendations are clearly described.  11. The health benefits, side effects, and risks have been considered in formulating the recommendations.  12. There is an explicit link between the recommendations and the supporting evidence.  13. The guideline has been externally reviewed by experts prior to its publication.  14. A procedure for updating the guideline is provided. | 7. Methods used to develop the classification scheme were reasonable and/or legitimate (and/or rigorous enough for the purpose) (i.e. narrative reviews can be reasonable, a systematic review is not essential).  6. The methods for developing the classification scheme are clearly described (i.e. did the authors clearly describe what they did, to the point where reproduction would be possible?).  8. The evidence to support the classification scheme is clearly described (any evidence is appropriate, research or otherwise; explanation of what the classification scheme was based on).  9. The classification scheme has been externally reviewed by experts prior to its publication (7 = CLASSIFICATION SCHEME is reviewed prior to publication by experts and PAPER is peer reviewed, 5 = PAPER is peer reviewed, but no mention of external/expert review of CLASSIFICATION SCHEME during development [E.g. external review could be sent to experts, Technical Expert Panel], 1 = no external review/peer review). | We wanted to ensure the methods were systematic, recognizing that methods, such as narrative reviews, are appropriate as a basis for classification schemes.  We wanted to ensure the methods for developing the classification schemes were clearly described and reproducible. One item in the adapted AGREE II tool covered two items (#8 and #10) in the original AGREE II tool.  The health benefits, side effects and risks (as per item #11 in the original AGREE II tool) were not relevant to classification schemes.  It was important to know what evidence the classification scheme was based on, but we were not as concerned about the strengths and limitations as per item #9 in the original AGREE II tool.  We were interested in knowing if the classification schemes were externally reviewed.  With so many classification schemes being adapted or extended, updating was less relevant in our measure of rigour. We assessed publication dates separately. |
| **Domain 4. Clarity of Presentation** | **Domain 4: Clarity of Presentation/Reporting** |  |
| 15. The recommendations are specific and unambiguous.  16. The different options for management of the condition or health issue are clearly presented.  17. Key recommendations are easily identifiable. | 10. The classification scheme is well described, unambiguous, specific. | This was important in assessing the usability/accessibility of the classification scheme.  #16 was not relevant to classification schemes  Similar to recommendations, the elements of the classification scheme should be easily identifiable, but this was covered under our item #10. |
| **Domain 5. Applicability** | **Domain 5: Applicability** |  |
| 18. The guideline describes facilitators and barriers to its application.  19. The guideline provides advice and/or tools on how the recommendations can be put into practice.  20. The potential resource implications of applying the recommendations have been considered.  21. The guideline presents monitoring and/or auditing criteria. | 11. The paper describes facilitators and barriers to the application of the classification scheme.  12. The paper describes advice and/or examples on how the classification scheme can be put into practice.  13. The potential resource implications (training, personnel) of applying the classification scheme have been considered.  14. The paper presents evaluation criteria for the classification scheme (could be monitoring/auditing, could be pre/post evaluation). | There may be known facilitators and barriers that will impact the application of a given classification scheme.  Any suggestions on how these schemes could be, or were used, was felt to increase their usefulness to researchers.  Any mention of resource implications, if noted, were felt to be relevant to their application by researchers.  As with guidelines, evaluating the application of classification schemes can facilitate their ongoing use. |
| **Domain 6: Editorial Independence** | **Domain 6: Editorial Independence** |  |
| 22. The views of the funding body have not influenced the content of the guideline.  23. Competing interests of guideline development group members have been recorded and addressed. | 15. The views of the funding body have not influenced the content of the classification scheme (Not influenced = 7, influenced = 1, Unclear = 4 [E.g. funding sources indicated but not clear if they influenced or not, or funding not indicated]).  16. Competing interests of the classification scheme development group members have been recorded and addressed [7=recorded and addressed; 1=not recorded or addressed; 2-6=aspects of recording competing interests and addressing them vary (E.g., competing interests recorded for some but not all contributors, some competing interests addressed, but not all)]. |  |
| **Overall Guideline Assessment** | **Overall Assessment** |  |
| 1. Rate the overall quality of this guideline.  1 (Lowest possible quality) to 7 (Highest possible quality)  2. I would recommend this guideline for use.  (Yes; Yes with modifications No)  Notes: | 1. Rate the overall quality of this paper/classification scheme (1=lowest possible quality to 7=highest possible quality [not just added/averaged, overall impressions and whether you would recommend]).  2. I would recommend this classification scheme for use (Yes; No).  Notes: | The “yes with modifications” category was not used in the overall assessment because modifications have already been made, in some cases, to the classification schemes; and further modifications are possible depending on the purposes of the researcher. We report adaptability elsewhere (see Table 3). |
